# Origin of purple asparagus cultivar ‘Pacific Purple’ based on the sequence of sex determination gene

**DOI:** 10.3389/fpls.2023.1237433

**Published:** 2023-11-16

**Authors:** Akira Kanno, Nana Hirobe, Lei Zhang

**Affiliations:** Graduate School of Life Sciences, Tohoku University, Sendai, Japan

**Keywords:** sex determination gene, *MSE1/AoMYB35/AspTDF1*, Violetto d’Albenga, purple asparagus, sex identification marker

## Abstract

Garden asparagus is one of the most important crops worldwide. Since this crop is dioecious and male plants generally have higher yields compared to female plants, several DNA markers for sex identification have been developed for acceleration of asparagus breeding. Among these markers, Asp1-T7sp and MSSTS710 were found to be effective in sex determination for many asparagus cultivars. However, we previously found that these markers were not completely suitable for sex identification in the purple asparagus cultivar ‘Pacific Purple’. There are two types of male individuals in this cultivar: One type is PP-m, which is identified the sex type by Asp1-T7sp and MSSTS710 markers, while the other type is PP-m* whose sex type is not identified by these markers. Since the sex identification markers are located on the non-recombining Y region, it was expected that the sequence around this region might be different between PP-m and PP-m*. In this study, the sequence of one of the sex-determining genes, *MSE1/AoMYB35/AspTDF1*, was analyzed, and a comparative analysis was conducted among PP-m and PP-m* of ‘Pacific Purple’, *A. officinalis* and related species *A. maritimus*. The results revealed that PP-m and PP-m* has the similar sequence of *MSE1/AoMYB35/AspTDF1* gene from *A. officinalis* and *A. maritimus*, respectively. ‘Pacific Purple’ is a cultivar developed through polycross hybrid from Italian landrace ‘Violetto d’Albenga’ (VA), suggesting that VA originated from an interspecific crossing between *A. officinalis* and *A. maritimus* and that the pollen parent used in ‘Pacific Purple’ breeding contained two types of male individuals with different *MSE1/AoMYB35/AspTDF1* sequence. As a result, PP-m and PP-m* of ‘Pacific Purple’ harbors the similar sequences of the *MSE1/AoMYB35/AspTDF1* gene from *A. officinalis* and *A. maritimus*, respectively.

## 1 Introduction

Garden asparagus (*Asparagus officinalis* L.) is one of the most economically important crops and cultivated in many countries. This species belongs to the genus *Asparagus* (Asparagaceae), which is a member of the Asparagaceae (APG III, 2009). The genus *Asparagus* includes over 200 species (Kanno and Yokoyama, 2011) and contains dioecious and hermaphrodite species (Kubota et al., 2012). Garden asparagus is a dioecious species, like *A. kiusianus* and *A. maritimus*, and male and female flowers were produced on male and female plants, respectively. The sex of this species is determined by sex chromosomes, X and Y, and males are heterogametic [XY], and females are homogametic [XX] (Rick and Hanna, 1943; Harkess et al., 2017). The two sex chromosomes are cytologically homomorphic (Löptien, 1979) and the genotype of males and females is also shown as [*Mm*] and [*mm*], respectively (Rick and Hanna, 1943; Sneep, 1953). Whole-genome sequencing of garden asparagus revealed the existence of around 1Mb non-recombining male-specific region on Y chromosome (Harkess et al., 2017). There are 13 genes located on this non-recombining Y region and two of them were detected as sex determination genes (Harkess et al., 2017). One of the gene is *SOFF* which is responsible for suppressing female organogenesis (Harkess et al., 2017; Harkess et al., 2020) and the other gene is *MSE1/AoMYB35/AspTDF1* for stamen development (Harkess et al., 2017; Murase et al., 2017; Tsugama et al., 2017).

There are almost no morphological differences between male and female plants during the vegetative growth phase, and the morphological differences are only observed in floral organs (Marziani Longo et al., 1990; Marziani et al., 1999). Since garden asparagus takes 1-2 years from germination to flowering, several sex-specific DNA markers such as MSSTS710 and Asp1-T7sp have been developed to speed up the asparagus breeding process (Nakayama et al., 2006; Kanno et al., 2014). MSSTS710 and Asp1-T7sp markers were shown to be effective for sex determination in many cultivars of garden asparagus (Nakayama et al., 2006; Kanno et al., 2014). MSSTS710 is only available for use in *A. officinalis* and cannot be used for sex identification in other *Asparagus* species, while Asp1-T7sp has been shown to be available in garden asparagus and some related *Asparagus* species (Nakayama et al., 2006; Kubota et al., 2012; Kanno et al., 2014; Kanno et al., 2020). This marker is also applicable in ‘Morado de Huetor’ (MH), which is tetraploid landrace in Spain (Regalado et al., 2014; Regalado et al., 2016).

Using these markers, the sex of asparagus can be identified during seedling stage. Thus, we applied these DNA markers for analyzing the sex difference of the yield of green and purple asparagus cultivars in our recent report (Motoki et al., 2022). We used two cultivars of green asparagus, ‘Early California’ and ‘UC157’, as well as one cultivar of purple asparagus, ‘Pacific Purple’ for this analysis. We identified the sex of over 50 individuals for each cultivar by MSSTS710 and Asp1-T7sp markers, planted them and compared the yield differences between male and female asparagus plants (Motoki et al., 2022). After flowering, we found that the sex genotype of two green asparagus cultivars was completely identified by MSSTS710 and Asp1-T7sp markers, however, some male individuals of ‘Pacific Purple’ were detected as “female” by these markers. This indicated that ‘Pacific Purple’ is heterogeneous cultivar and this cultivar has two types of male individuals: one type is identified the sex by MSSTS710 and Asp1-T7sp markers and the other type is not (Mitoma et al., 2018).

‘Pacific Purple’ is a tetraploid purple cultivar. This cultivar, as well as ‘Purple Passion’, was obtained from ‘Violetto d’Albenga’ (VA), which is tetraploid landrace in Italy (Benson et al., 1996; Falloon and Andersen, 1999). Moreno et al. (2006) performed RAPD analysis to clarify the phylogenetic relationship among *A. officinalis*, ‘Purple Passion’ and ‘Morado de Huetor’ (MH), which is another tetraploid landrace in Spain. They found that MH and ‘Purple Passion’ were well differentiated from *A. officinalis* (Moreno et al., 2006). Their research group also estimated the origin of VA and MH using phylogenetic analysis based on the sequence of the ITS region (Moreno et al., 2008a). Their results indicated that VA belongs to the same clade as *A. officinalis*, while MH is likely an interspecific hybrid between *A. officinalis* and *A. maritimus* (Moreno et al., 2008a). This suggests that VA might have originated from cross hybridization between *A. officinalis* and related species such as *A. maritimus*, but the evolutionary history was different from MH. Since ‘Pacific Purple’ is polycross hybrid bred from VA, which is likely to be an interspecific hybrid between *A. officinalis* and *A. maritimus*, this might be the cause of heterogeneity of this cultivar (Mitoma et al., 2018).

In our previous report, we designated the two types of male individuals found in ‘Pacific Purple’ as PP-m and PP-m*: PP-m is the strain which is identified the sex by MSSTS710 and Asp1-T7sp markers and PP-m* is the strain whose sex type is not identified by these markers (Mitoma et al., 2018). We developed two additional markers, MspHd and AspMSD, based on the sequence of Asp1-T7sp marker region and *MSE1/AoMYB35/AspTDF1* gene region, respectively. AspMSD marker could be applied for sex identification to both PP-m and PP-m* male individuals, although MspHd marker could be applicable in only PP-m individuals (Mitoma et al., 2018). The difference of the applicability of the sex marker in PP-m and PP-m* might be due to some mutations or rearrangements around the male specific region on the Y chromosome (Mitoma et al., 2018). In this study, we aimed to obtain better understanding of the heterogeneity found in ‘Pacific Purple’. Here we analyzed the differences between PP-m and PP-m* by revealing the sequences of the sex-determining marker regions on the non-recombining Y region to clarify the reason why these two types of male individuals are coexisting in ‘Pacific Purple’. Based on the results, we discussed the reasons for the occurrence of PP-m and PP-m* during the breeding process of ‘Pacific Purple’ from the Italian landrace VA.

## 2 Materials and methods

### 2.1 Plant materials

The following plant materials were used in this study: *A. officinalis* ‘Mary Washington 500W’; purple asparagus cultivar ‘Pacific Purple’, ‘Purple Passion’, NJ1064 (an all-male strain), and ‘Erasmus’ (an all-male cultivar, provided by Bejo Japan KK); and *A. maritimus*. The list of these samples were shown in **Table 1**. These plants were cultivated in a greenhouse at the Graduate School of Life Sciences, Tohoku University, Japan.

**Table 1:** The list of the samples used for the sequence and genetic analysis in this study.

Line	Species	Cultivar/Strain	Sex
AO0011M	*Asparagus officinalis*	‘Mary Washinton 500W’	Male
AO0012M	*Asparagus officinalis*	‘Mary Washinton 500W’	Male
MM001F	purple asparagus	‘Pacific Purple’	Female
MM003M	purple asparagus	‘Pacific Purple’	Male
MM004M	purple asparagus	‘Pacific Purple’	Male
MM018F	purple asparagus	‘Pacific Purple’	Female
MM029M	purple asparagus	‘Pacific Purple’	Male
MM032M	purple asparagus	‘Pacific Purple’	Male
MM060M	purple asparagus	‘Pacific Purple’	Male
BD078M	purple asparagus	‘Purple Passion’	Male
BD081M	purple asparagus	‘Purple Passion’	Male
ER001M	purple asparagus	‘Erasmus’	Male
ER002M	purple asparagus	‘Erasmus’	Male
NJ023M	purple asparagus	NJ1063	Male
NJ063M	purple asparagus	NJ1063	Male
ASB001M-1	*Asparagus maritimus*		Male
ASB002M-1	*Asparagus maritimus*		Male
AC0101M	*Asparagus cochinchinensis*		Male

### 2.2 PCR analysis using sex identification markers and indel specific primers

Total DNA was extracted from asparagus cladodes as described previously (Honda and Hirai, 1990). For PCR analysis, two sets of male-specific primers were used: AspMSD-fw and AspMSD-rv, and MspHd-fw and MspHd-rv published in Mitoma et al. (2018). These primer sets amplify 0.55 kb and 1.1 kb fragments, respectively. AODEFint4fw and AODEFint4rv, which amplify 0.5 kb of the intron of *AODEF* gene, was used for positive control as described previously (Kubota et al., 2012). For indel specific PCR, we used MSE1-InDel-fw and MSE1-InDel-rv primers. The primers used in this study were listed in **Table S1**.

PCR was performed in a volume of 25 μl reaction mixture containing 50 ng total DNA and 50 pmol each primer using Quick Taq HS DyeMix (Toyobo, Osaka, Japan) with a TaKaRa PCR Thermal Cycler Dice (TaKaRa, Shiga, Japan). The PCR reactions were denatured for 2 min at 94°C, followed by 30 cycles of 30 sec at 94°C, 30 sec at 58°C, and 1 min at 72°C, with a final cycle of 72°C for 10 min. Subsquently, gel electrophoresis for the amplified DNA fragments were performed with 0.9% agarose gels in 1×TAE buffer. After stained with ethidium bromide, the gels were observed and photographed under UV light.

### 2.3 Isolation of *MSE1/AoMYB35/AspTDF1* gene from *A. officinalis*, purple asparagus cultivars, and *A. maritimus*

Total DNA was extracted from cladodes as described above (Honda and Hirai, 1990). MSE1-fw and MSE1-rv were used for cloning the full-length of genomic clone of *AspTDF1/MSE1* gene. The primers used in this study were listed in **Table S1**. For genomic PCR, TaKaRa ExTaq polymerase (TaKaRa, Japan) was used in a volume of 25 µl reaction mixture containing 50 ng of total DNA, 0.2 mM of each dNTPs, 1 × Ex Taq buffer, 0.5 units of TaKaRa ExTaq polymerase, and 0.5 µM of each primer with a TaKaRa PCR Thermal Cycler Dice (TaKaRa). The PCR consisted of an denaturation step for 2 min at 96°C, followed by 30 cycles of 30 sec at 96°C, 30 sec at 60°C, and 2.5 min at 72°C, with a final cycle of 72°C for 10 min. The PCR products were cloned into pGEM-T Easy vector (Promega, USA) and sequenced using an Applied Biosystems Big Dye Terminator V3.1 (Applied Biosystems, Carlsbad, CA, USA), according to the manufacturer’s instructions.

### 2.4 Phylogenetic analysis of *MSE1/AoMYB35/AspTDF1* gene

Phylogenetic analysis was conducted using MEGA11 software (Tamura et al., 2021). Full-length predicted amino acid sequences and nucleotide sequences were aligned by MEGA. The phylogenetic tree was constructed using Neighbor joining method and a bootstrap consensus tree was inferred from 1,000 replicates (Felsenstein, 1985).

## Results

### 3.1 Sex identification with AspMSD and MspHd markers

**Figure 1:**
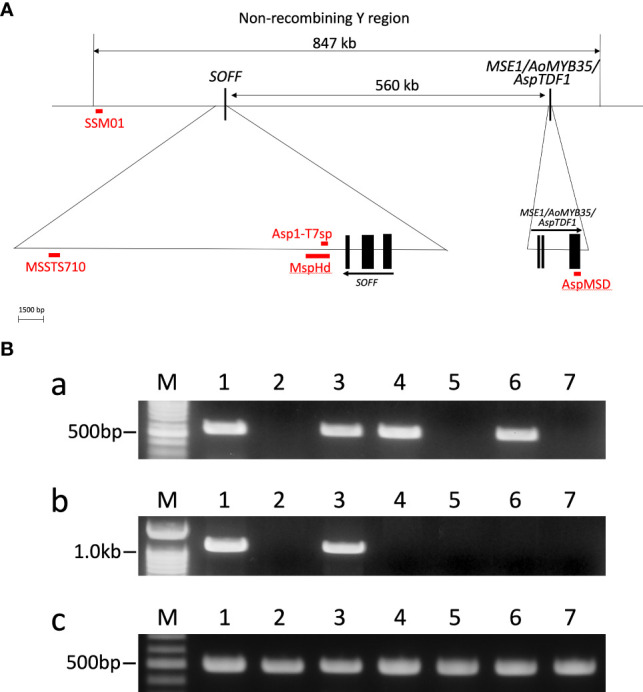
**(A)** Positions of sex identification markers around non-recombining Y region. The solid squares showed the exons of the two sex determination genes. Arrows indicate the direction of transcription of the gene. This figure is based on Mitoma et al. (2018). **(B)** PCR analysis of *A. officinalis*, purple asparagus and *A. maritimus* using male-specific AspMSD (a) and MspHd (b) primers, and AODEFint4 primer (c) for positive control (Kubota et al., 2012). Lane 1: *A. officinalis* male, Lane 2: *A. officinalis* female, Lane 3: ‘Pacific Purple’ male (PP-m), Lane 4: ‘Pacific Purple’ male (PP-m*), Lane 5: ‘Pacific Purple’ female, Lane 6: *A. maritimus* male, Lane 7: *A. maritimus* female. Lane M: 100 bp ladder markers.

To date, several dominant sex identification markers, such as AspMSD, MSSTS710, Asp1-T7sp and MspHd, have been developed and all of these markers were applicable to identify the sex of many cultivars in *A. officinalis* (**Figure 1A**; Nakayama et al., 2006; Kanno et al., 2014; Mitoma et al., 2018). Among these markers, the applicability of AspMSD, MSSTS710 and Asp1-T7sp markers were analyzed in other *Asparagus* species (Kubota et al., 2012; Mitoma et al., 2018; Kanno et al., 2020). Here we analyzed the applicability of MspHd marker for the sex identification in PP-m and PP-m* of ‘Pacific Purple’, including *A. officinalis* and *A. maritimus.* Although the applicability of MspHd marker in ‘Pacific Purple’ was previously reported (Mitoma et al., 2018), we added *A. maritimus* for this analysis because RAPD analysis has revealed that VA might originated from cross hybridization between *A. officinalis* and related species such as *A. maritimus* (Moreno et al., 2008a).

Total DNA was extracted from male and female individuals of *A. officinalis*, *A. maritimus* and ‘Pacific Purple’ including two types of males (PP-m and PP-m*). PCR analysis with these total DNAs as template was performed using AspMSD and MspHd markers, as well as AODEFint4 primer set for positive control (Kubota et al., 2012). Using AspMSD marker, PCR products were observed in all male individuals of *A. officinalis*, *A. maritimus* and ‘Pacific Purple’, while no amplification was detected in female individuals of these species (**Figure 1B**). Meanwhile, PCR fragment was detected only in male individuals of *A. officinalis* and PP-m and there was no amplification in PP-m*, male of *A. maritimus* and all female individuals by MspHd marker (**Figure 1B**). The applicability of other sex identification markers, MSSTS710 and Asp1-T7sp, in *Asparagus* species have already been reported (Kanno et al., 2014; Mitoma et al., 2018; Kanno et al., 2020), and these data were summarized in **Table 2**.

**Table 2:** Applicability of the dominant markers for sex identification in *A. officinalis*, ‘Pacific Purple’ and *A. maritimus*.

Species/cultivars	MSSTS710	MspHd	Asp1-T7sp	AspMSD
*A. officinalis*	+	+	+	+
Pacific Purple’ (PP-m)	+	+	+	+
Pacific Purple’ (PP-m*)	–	–	–	+
*A. maritimus*	–	–	+	+

+, applicable; -, not applicable.

### 3.2 Structural and phylogenetic analysis of the MSE1 gene

**Figure 2:**
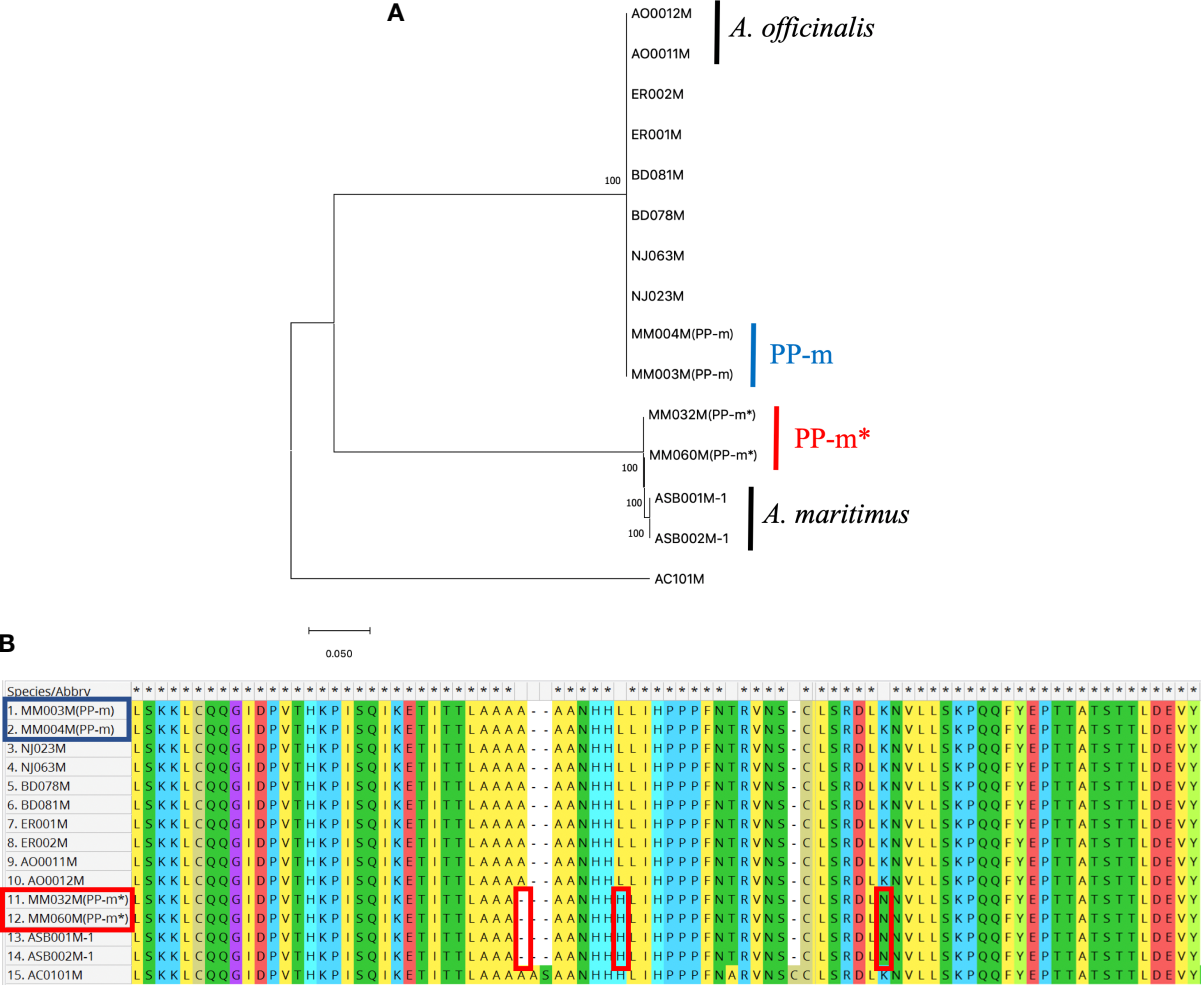
**(A)** Phylogenetic analysis of amino acid sequences of *MSE1/AoMYB35/AspTDF1* gene from *A. officinalis*, purple asparagus, *A. maritimus*. Values above branches represent bootstrap values (1,000 replicates). **(B)** The alignment of partial amino acid sequence of *MSE1/AoMYB35/AspTDF1* gene from *A. officinalis*, purple asparagus, *A. maritimus*. The red squares show the difference between *A. officinalis*/PP-m and *A. maritimus*/PP-m*. The strain names in the red square are the PP-m* sequences and in blue those of PP-m.

In order to analyze the genetic polymorphism in the non-recombining Y region of PP-m and PP-m*, AspMSD marker region can be used to compare the sequences among *A. officinalis*, *A. maritimus* and ‘Pacific Purple’. Since AspMSD marker was created based on the sequence of *MSE1/AoMYB35/AspTDF1* gene, the genomic sequence of this gene was analyzed among several purple asparagus cultivars as well as *A. officinalis* and *A. maritimus*. Total DNAs were extracted from two male individuals of each purple asparagus cultivars (PP-m and PP-m* of ‘Pacific Purple’, ‘Purple Passion’, ‘Erasmus’ and NJ1064), *A. officinalis* and *A. maritimus.* We also used *A. cochinchinensis* for the outgroup of phylogenetic analysis and these samples were listed in **Table 1**. After PCR amplification with gene specific primers, amplified fragments were cloned into plasmid vector and sequenced. To understand the phylogenetic relationship of *MSE1/AoMYB35/AspTDF1* gene among asparagus cultivars and related species shown above, deduced amino acid sequences were used to construct the phylogenetic tree. As shown in **Figure 2A**, PP-m* of ‘Pacific Purple’ belongs to the same clade of *A. maritimus*, and the other purple asparagus cultivars such as PP-m of ‘Pacific Purple’, ‘Purple Passion’, ‘Erasmus’ and NJ1064 were in the same clade of *A. officinalis*. This indicates that the non-recombining Y region of PP-m and PP-m* from ‘Pacific Purple’ is likely to have a high homology with that from *A. officinalis* and *A. maritimus*, respectively. Furthermore, comparison of deduced amino acid sequences showed that the *MSE1/AoMYB35/AspTDF1* sequence from PP-m* of ‘Pacific Purple’ was very similar to that from *A. maritimus* (or its closely related species) and that gene from the other purple asparagus cultivars was very similar to that from *A. officinalis* (**Figure 2B**).

**Figure 3:**
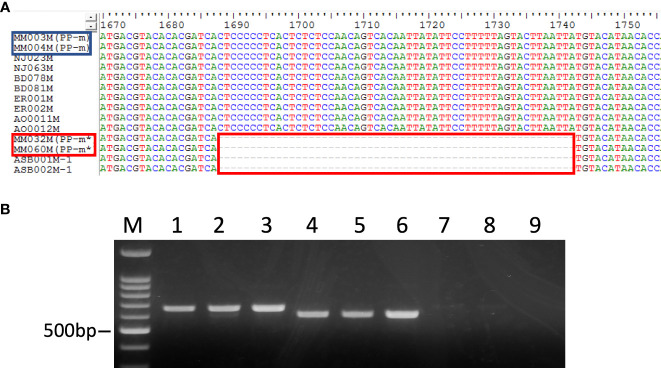
**(A)** The alignment of partial nucleotide sequence of *MSE1/AoMYB35/AspTDF1* gene from *A. officinalis*, purple asparagus, *A. maritimus*. The red squares show the difference between *A. officinalis*/PP-m and *A. maritimus*/PP-m*. The strain names in the red square are the PP-m* sequences and in blue those of PP-m. **(B)** PCR analysis of purple asparagus ‘Pacific Purple’ using MSE1-InDel primers. Lane 1-3: male (PP-m), lane 4-6: male (PP-m*), lane 7-9: female. Lane M: 100 bp ladder marker.

In addition, the insertion and deletion (indel) was observed in the intron region of the *MSE1/AoMYB35/AspTDF1* gene (**Figure 3A**). The size of indel was 55bp and the intron size of PP-m* of ‘Pacific Purple’ and *A. maritimus* was shorter than that of PP-m and *A. officinalis.* The size and the position of indel was completely the same in PP-m* of ‘Pacific Purple’ and *A. maritimus*, and also in PP-m and *A. officinalis* (**Figure 3A**). To analyze this indel polymorphism among ‘Pacific Purple’, we isolated total DNAs from 13 PP-m male individuals, 26 PP-m* individuals and 6 female individuals of ‘Pacific Purple’. PCR amplification was performed with the primers which amplify the indel region. As shown in **Figure 3B**, one PCR fragment was detected in PP-m and PP-m* and the size of the fragment of PP-m* is shorter than that of PP-m, while no PCR fragment was detected in female.

### 3.3 Sex ratio of ‘Pacific Purple’

In order to detect the sex ratio of ‘Pacific Purple’, male individuals of PP-m (MM029M) and PP-m* (MM060M) were selected to cross with female individuals (MM001F and MM018F). The sex of the progeny generated these crosses were analyzed with AspMSD marker. As shown in **Table 3**, we got 92 progeny from MM018F x MM060M (PP-m*) and the number of male and female was 52 and 40, respectively. From MM001F x MM029M (PP-m), we got 105 progeny and the number of male and female was 62 and 43, respectively. This result showed that the sex ratio of ‘Pacific Purple’ was male:female = 1:1.

**Table 3:** Segregation of plant sex in the progenies obtained from crossing between female and male (PP-m/PP-m*) of ‘Pacific Purple’.

Cross	Male	Female	Total	χ^2^_1:1_
MM018F x MM060M (PP-m*)	52	40	92	0.21
MM001F x MM029M (PP-m)	62	43	105	0.06

There were no statistically significant differences between the ratio of male: female observed and the ratio expected when tested with χ2, α=5%.

## 4 Discussion

### 4.1 Applicability of sex identification markers

We analyzed the applicability of sex identification markers among *A. officinalis*, *A. maritimus*, and ‘Pacific Purple’ (**Figure 1B**; **Table 2**). AspMSD marker is applicable for all of these species, while MspHd and MSSTS710 were applicable in *A. officinalis* and PP-m of ‘Pacific Purple’, and not in PP-m* and *A. maritimus*. These results indicated that the sequence homology of these marker regions is different, although these markers are located on the non-recombining Y region (**Figure 1A**). AspMSD marker was generated using the exon sequence of *MSE1/AoMYB35/AspTDF1* gene (**Figure 1A**; Mitoma et al., 2018). Therefore, the sequence within the *MSE1/AoMYB35/AspTDF1* gene is probably conserved among *A. officinalis*, *A. maritimus* and ‘Pacific Purple’. On the other hand, the sequence around MspHd and MSSTS710 markers, which are located on the intergenetic regions, do not appear to be conserved.

It is interesting to note that the applicability of Asp1-T7sp marker is different in PP-m* of ‘Pacific Purple’ and *A. maritimus*: Asp1-T7sp marker is applicable in *A. maritimus* but not in PP-m* (**Table 2**). Asp1-T7sp marker can be used to identify the sex of various cultivars of *A. officinalis* and several dioecious *Asparagus* species, such as *A. kiusianus*, *A. maritimus*, *A. pseudoscaber* and *A. schoberioides* (Nakayama et al., 2006; Kubota et al., 2012; Kanno et al., 2020). Furthermore, this marker is applicable in ‘Morado de Huetor’ (MH), which is tetraploid landrace in Spain (Regalado et al., 2014; Regalado et al., 2016). ‘Pacific Purple’ was bred from VA, tetraploid landrace in Italy, and VA and MH are likely an interspecific hybrid between *A. officinalis* and *A. maritimus* (Falloon and Andersen, 1999; Moreno et al., 2008a). In addition, Asp1-T7sp marker region is located on just downstream of *SOFF* gene (**Figure 1A**). This indicated that the sequence around Asp1-T7sp marker is well conserved among the cultivars of *A. officinalis* and related species including *A. maritimus*. Since Asp1-T7sp marker cannot amplify the male-specific fragment in PP-m*, there might be some mutation and/or rearrangement occurred at the primer sequence of Asp1-T7sp marker in PP-m* of ‘Pacific Purple’. Difference of the applicability of Asp1-T7sp and MspHd markers for *A. maritimus* is also interesting because the position of Asp1-T7sp marker is located within MspHd marker region (**Figure 1A**; Mitoma et al., 2018). Further analysis comparing the sequence around MspHd marker region is necessary to clarify the genetic variation of the non-recombining Y region among ‘Pacific Purple’, *A. officinalis* and *A. maritimus.*

Recently, we developed SSM01 marker, which is able to distinguish between X and Y sex genotypes among *A. officinalis*, *A. maritimus* and ‘Pacific Purple’, including PP-m and PP-m* (Akahori and Kanno, 2022). The sequence of SSM01 marker region have previously been determined in these *Asparagus* species (Akahori and Kanno, 2022). However, due to the high similarity of their sequences, their phylogenetic relationship could not be clarified. SSM01 marker is located at the end of the non-recombining Y region although this marker is far from two sex determination genes (**Figure 1A**; Akahori and Kanno, 2022). Highly conserved SSM01 region may have some function in asparagus, but further analysis is needed.

### 4.2 Genotype of purple asparagus cultivar ‘Pacific Purple’

In diploid asparagus, the genotype of male and female is known as [*Mm*] and [*mm*], respectively (Rick and Hanna, 1943; Sneep, 1953). On the other hand, the report for the genotype of tetraploid asparagus is limited. The tetraploid Spanish landrace MH is well analyzed and the male and female genotypes of this population are [*Mmmm*] and [*mmmm*], respectively (Moreno et al., 2008b). The Italian landrace VA and purple asparagus cultivar derived from VA are known as tetraploid (Falloon and Andersen, 1999), however, the genotype of these population is not known well. Here we analyzed the sex ratio of ‘Pacific Purple’, and this cultivar showed male:female=1:1 (**Table 3**). Furthermore, the indel polymorphism observed in the *MSE1/AoMYB35/AspTDF1* gene showed that a single type of PCR fragment of different size is amplified when using PP-m and PP-m* DNAs as templates (**Figure 3B**). Our results lead us to assume that the male and female genotypes of ‘Pacific Purple’ is likely to be [*Mmmm*] and [*mmmm*], respectively.

### 4.3 Origin of PP-m and PP-m* in ‘Pacific Purple’

In this study, we determined the *MSE1/AoMYB35/AspTDF1* sequence from *A. officinalis*, *A. maritimus* and ‘Pacific Purple’. Phylogenetic analysis based on the amino acid sequences of this gene showed that PP-m* was closely related to *A. maritimus* and PP-m grouped in the same clade of *A. officinalis* (**Figure 2A**). The comparison of the amino acid sequence and the indel found in the intron of the *MSE1/AoMYB35/AspTDF1* gene among these species showed that the *MSE1/AoMYB35/AspTDF1* sequence from PP-m and PP-m* was very similar to that from *A. officinalis* and *A. maritimus*, respectively (**Figures 2B**, **3A**). These results revealed that *MSE1/AoMYB35/AspTDF1* gene from PP-m and PP-m*of ‘Pacific Purple’ was derived from *A. officinalis* and *A. maritimus*, respectively. This nicely fit with the results of the applicability of sex determination markers (**Table 2**). MSSTS710 and MspHd markers were available for *A. officinalis* and PP-m of ‘Pacific Purple’, but not for *A. maritimus* and PP-m* of ‘Pacific Purple’, suggesting that the non-recombining Y region of PP-m and PP-m* have high homology with that of *A. officinalis* and *A. maritimus*, respectively.

The purple asparagus cultivar ‘Pacific Purple’ was developed using the Italian landrace VA. This cultivar is a polycross hybrid and its offspring from the polycross block showed a uniform purple color (Falloon and Andersen, 1999). This indicates that multiple parents were used for the breeding of ‘Pacific Purple’. Based on the result of *MSE1/AoMYB35/AspTDF1* sequence, PP-m has the same *MSE1/AoMYB35/AspTDF1* gene sequence as *A. officinalis*, and PP-m* has the same as *A. maritimus*. In addition, the male genotype of ‘Pacific Purple’ is likely to be [*Mmmm*] as shown above. These results indicate that two types of pollen parents with different *MSE1/AoMYB35/AspTDF1* genes were included in the polycross block used for the breeding of ‘Pacific Purple’. This is consistent with the speculation that VA is likely an interspecific hybrid between *A. officinalis* and *A. maritimus* (Moreno et al., 2008a).

**Figure 4:**
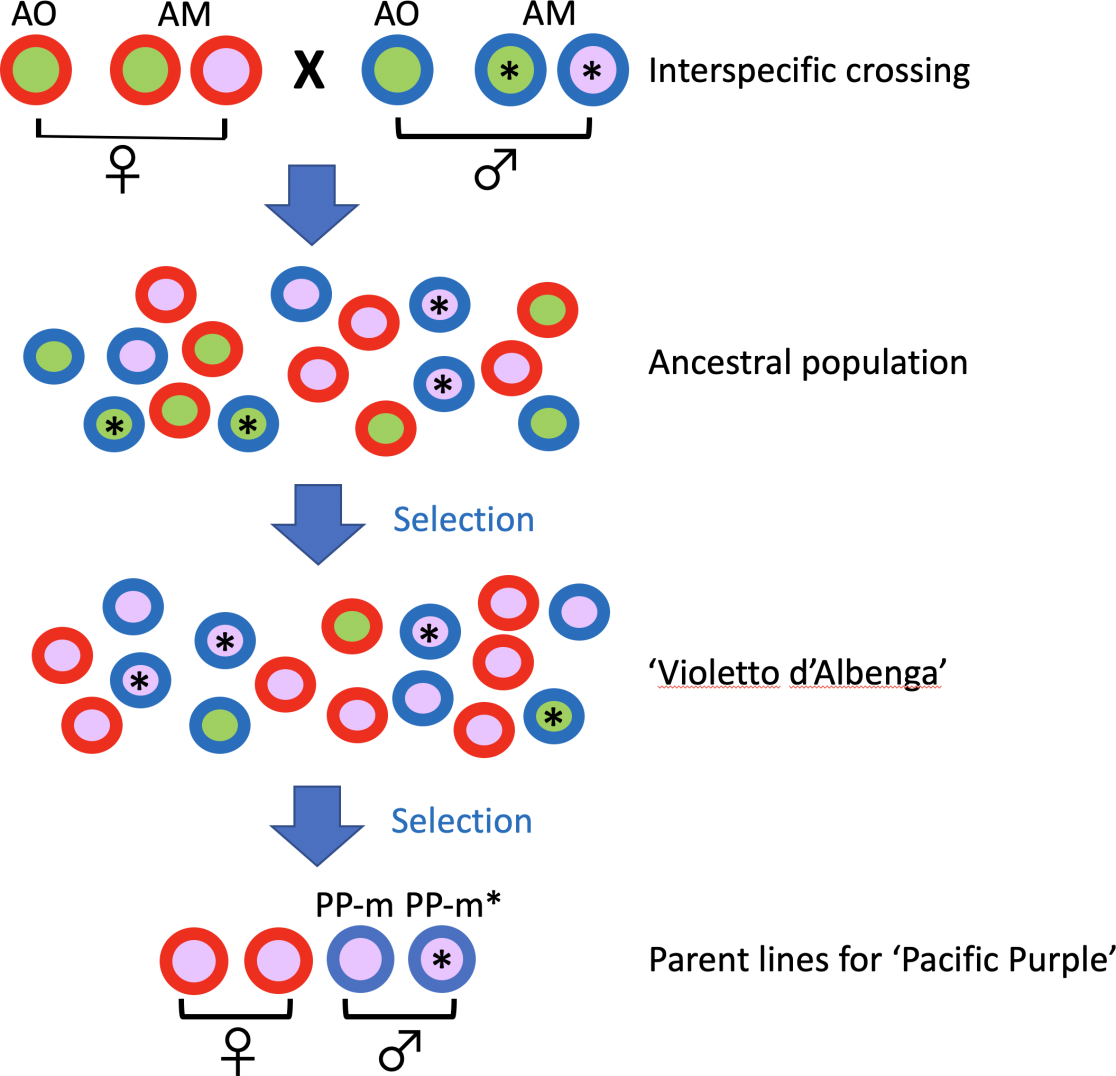
Schematic diagram showing the breeding process of ‘Pacific Purple’. Male individuals are represented by circles with blue rims, while female individuals are shown with red rims. Male individuals with an asterisk (*) possess the *A. maritimus*/PP-m type of *MSE1/AoMYB35/AspTDF1*, while those without an asterisk have the *A. officinalis*/PP-m type. The circle colors, green and purple, indicate spear color.

Based on our results, the putative breeding process of ‘Pacific Purple’ is shown in **Figure 4**. Since ‘Pacific Purple’ is a cultivar developed from VA (Falloon and Andersen, 1999), it is expected that ancestral population of VA had maintained the interspecific crossing between *A. officinalis* and *A. maritimus*. During the selection process of VA from ancestral population, two types of pollen parents with different non-recombining Y region might have been maintained in their population. At the final step of pollen parent selection of ‘Pacific Purple’, the breeder selected two types of males with different *MSE1/AoMYB35/AspTDF1* sequence, resulting that two types of male, i.e. PP-m and PP-m*, were included in ‘Pacific Purple’.

On the other hand, other purple asparagus cultivars/lines of ‘Purple Passion’, ‘Erasmus’, NJ1064, have one type of *MSE1/AoMYB35/AspTDF1* sequence of *A. officinalis*/PP-m type (**Figures 2**, **3**). Among these purple asparagus cultivars, at least ‘Purple Passion’ has been shown to be derived from VA, like ‘Pacific Purple’ (Benson et al., 1996). ‘Purple Passion’ was bred from single plant selection, while ‘Pacific Purple’ bred from polycross hybrid (Benson et al., 1996; Falloon and Andersen, 1999). This is consistent with the result that only the *A. officinalis*/PP-m type of *MSE1/AoMYB35/AspTDF1* sequence was obtained from ‘Purple Passion’.

In conclusion, PP-m and PP-m* of ‘Pacific Purple’ has the similar sequence of *MSE1/AoMYB35/AspTDF1* gene from *A. officinalis* and *A. maritimus*, respectively. ‘Pacific Purple’ was bred from VA, which is likely to be developed from an interspecific crossing between *A. officinalis* and *A. maritimus*. Since ‘Pacific Purple’ was a polycross hybrid, two types of pollen parents with different *MSE1/AoMYB35/AspTDF1* sequence is likely to be used for the breeding of this cultivar. Since several purple asparagus cultivars derived from VA are known to date, it is interesting to analyze the *MSE1/AoMYB35/AspTDF1* sequence of those cultivars.

## Data availability statement

The raw data supporting the conclusions of this article will be made available by the authors, without undue reservation.

## Author contributions

Experimental design: AK. Experiments: AK, NH, LZ. Data analysis: AK, NH, LZ. Manuscript preparation: AK. Supervision, funding, and reagents: AK. All authors contributed to the article and approved the submitted version.
